# Supplementary figures and images for: Local Inflammatory Mediators Involved in Neuropathic Pain
Source: Int J Mol Sci. 2023 Apr 25;24(9):7814. doi: 10.3390/ijms24097814 (PMC10178336; doi:10.3390/ijms24097814)

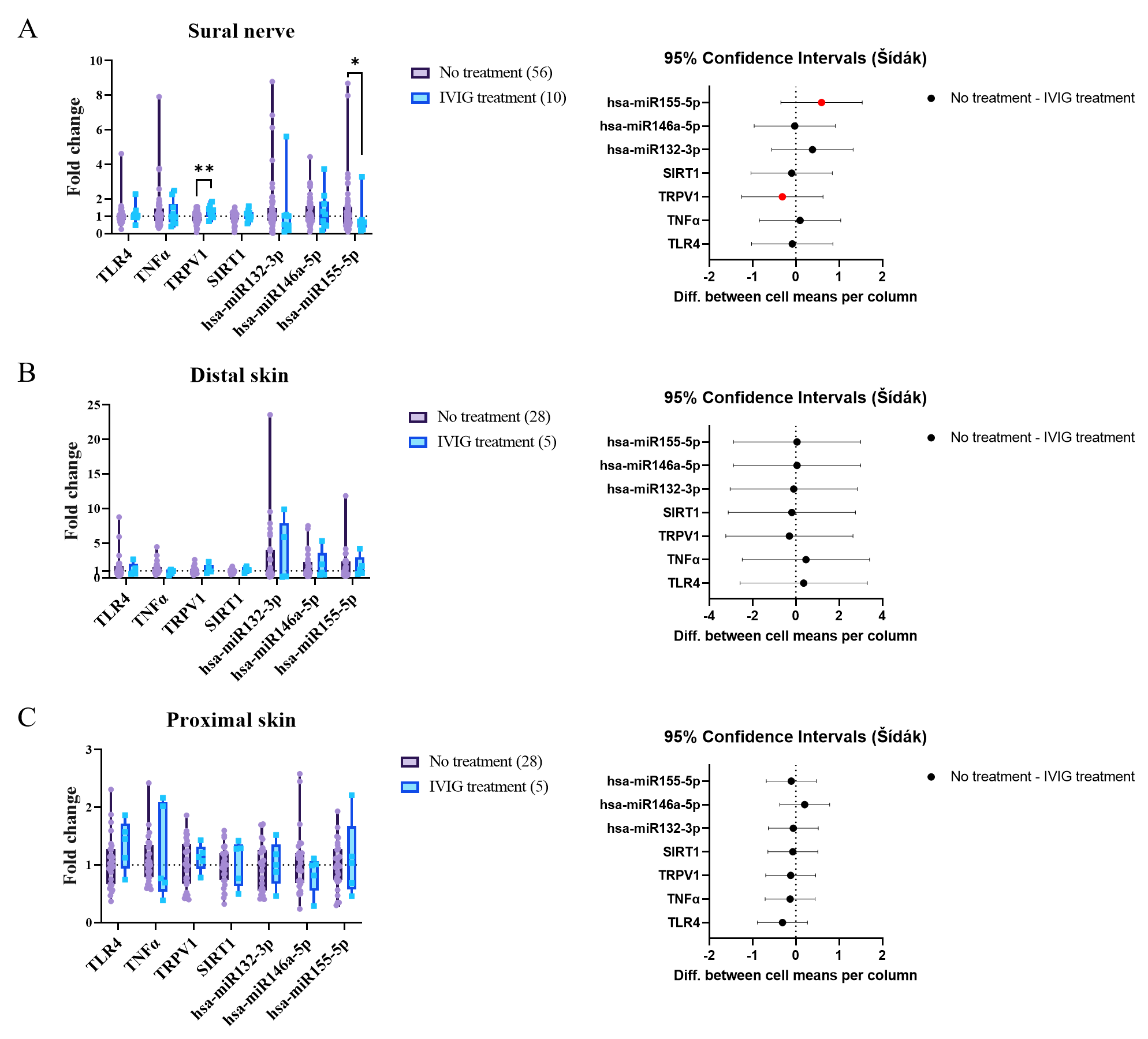

Supplement: Supplementary file 1 [file ijms-24-07814-s001.zip › ijms-2337887-supplementary.tif]
